# Outcomes of Acute Mesenteric Ischemia in End-Stage Renal Disease and Predictors of Mortality: A Nationwide Assessment

**DOI:** 10.7759/cureus.37657

**Published:** 2023-04-16

**Authors:** Vikash Kumar, Dhir Gala, Miranda Green, Mili Shah, Hamsika Moparty, Vijay Reddy Gayam, Praneeth Bandaru, Suut Gokturk, Madhavi Reddy, Vinaya Gadaputi

**Affiliations:** 1 Internal Medicine, The Brooklyn Hospital Center, Brooklyn, USA; 2 Internal Medicine, American University of the Caribbean School of Medicine, Sint Maarten, SXM; 3 Gastroenterology and Hepatology, The Brooklyn Hospital Center, Brooklyn, USA; 4 Gastroenterology and Hepatology, Blanchard Valley Health System, Findlay, USA

**Keywords:** ami, predictors of mortality, nationwide inpatient sample (nis), end stage renal disease (esrd), acute mesenteric ischemia

## Abstract

Background

Acute mesenteric ischemia (AMI) is an uncommon disease caused by obstruction of blood flow to the bowel, which can lead to high mortality rates. End-stage renal disease (ESRD) is another disease commonly seen in the elderly. There are limited data evaluating the relationship between AMI and ESRD, but it has been shown that ESRD patients have a higher risk of mesenteric ischemia than the general population.

Methods

This retrospective analysis utilized the National Inpatient Sample database for 2016, 2017, and 2018 to identify patients with AMI. Patients were then divided into two groups, AMI with ESRD and AMI only. All-cause in-patient mortality, hospital length of stay (LOS), and total costs were identified. The Student's t-test was used to analyze continuous variables, while Pearson's Chi-square test was used to analyze categorical variables.

Results

A total of 169,245 patients were identified, with 10,493 (6.2%) having ESRD. The AMI with ESRD group had a significantly higher mortality rate than the AMI-only group (8.5% vs 4.5%). Patients with ESRD had a longer LOS (7.4 days vs 5.3 days; P = 0.00), and higher total hospital cost ($91,520 vs $58,175; P = 0.00) compared to patients without ESRD.

Conclusion

The study found that patients with ESRD who were diagnosed with AMI had a significantly higher mortality rate, longer hospital stays, and higher hospital costs than patients without ESRD.

## Introduction

Mesenteric ischemia is a relatively uncommon disease that results from inadequate blood flow in the mesenteric circulation. Although the overall incidence of acute mesenteric ischemia (AMI) is low at about 0.2% of all acute admissions to the emergency department, the high mortality rate (50% to 80%) makes being able to recognize trends and promptly diagnose and treat these patients very important [1,2]. The overall mortality from AMI has been decreasing over the last few decades as treatment by open surgical repairs has been replaced with mesenteric angioplasty and stenting. A study in the USA from 2000 to 2012 found that annual population-based mortality for AMI decreased from 12.9 to 5.3 deaths per million/year [3]. These numbers could potentially continue declining with advancements in both treatment and diagnostic tools. High mortality rates in AMI are often associated with delayed treatment due to delayed diagnosis; therefore, prompt CT angiography, especially for elderly patients with any symptoms of AMI, is highly recommended [4]. Once diagnosed, management of AMI can include surgery, fluid resuscitation, antibiotic coverage, and therapeutic anticoagulation. Although an open transperitoneal approach via full midline incision in the abdomen is still considered the gold standard, as previously mentioned, more minimally invasive options are becoming common. Treatments include thromboembolectomy, bypass, and endovascular therapy. These can be performed open or percutaneously. Percutaneous treatments have been shown to lower in-hospital mortality, length of stay (LOS), and bowel resection significantly when compared to open surgical repair [5].

Another disease more commonly seen in the elderly is an end-stage renal disease (ESRD). The prevalence of ESRD continues to increase in the United States [6]. ESRD occurs when one’s kidneys fail to work at the level necessary to keep them alive. The cut-off is usually considered at less than 15% of typical kidney function [7]. For the treatment of ESRD, a kidney transplant tends to yield the best outcomes, but most cases are treated with dialysis. Controlling blood pressure and preserving peripheral veins in patients receiving dialysis also improves mortality in those with ESRD [8]. The exact pathophysiology of nonocclusive mesenteric ischemia in ESRD is poorly understood but it has been shown to be associated with patients on hemodialysis and those with ESRD [9]. There are limited data evaluating the relationship between AMI and ESRD; however, one study found that the risk of mesenteric ischemia for ESRD patients is 44.1 times higher than the general population and peritoneal dialysis treatment seems to have a higher associated risk of mesenteric ischemia than hemodialysis [10]. However, there is also a documented association between nonocclusive mesenteric ischemia and hemodialysis, mainly in older patients who spend more time on dialysis [11]. In addition, hemodialysis has been demonstrated to affect mesenteric circulation, such as increasing diameter, decreasing velocity, and volume flow [12]. More research is needed on this topic, but the correlation is evident. This study aims to further clarify the connection between these two by analyzing the effect of ESRD on the morbidity and/or mortality of patients diagnosed with AMI.

## Materials and methods

Study data

In conducting this retrospective analysis, we used data from the Healthcare Cost and Utilization Project's National Inpatient Sample (NIS), which is sponsored by the Agency for Healthcare Research and Quality (HCUP). With information on over seven million hospitalizations (unweighted), NIS is the largest publicly accessible all-payer administrative database; when weighted, it represents about 35 million hospitalizations nationally. Data for specific patients, doctors, and hospitals are safeguarded while information on clinical and resource utilization is provided. In order to reflect hospital systems' adoption of the International Classification of Diseases, Tenth Edition, Clinical Modification/Procedure Coding System (ICD-10-CM/PCS), the NIS began using it in October 2015 by implementing the ICD-10-CM/PCS. National estimates of the entire hospitalized population in the United States were calculated using the sampling and weighting method developed by the Agency for Healthcare Research and Quality.

Study design

A retrospective analysis was performed by utilizing the National Inpatient Sample Database from the years 2016, 2017, and 2018 and the International Classification of Diseases, Tenth Revision codes to identify patients > 18 years old with the principal diagnosis of AMI. We identified all-cause in-patient mortality, hospital LOS, and total costs by dividing the total population into two groups: AMI with ESRD and AMI only. For baseline characteristics, we used patient demographics (age, race, and sex), the Charlson Comorbidity Index, insurance status, hospital characteristics, and relevant comorbidities. Categorical variables were compared using the chi-square test, and continuous variables were compared using the t-test. Confounding variables were adjusted using multivariate logistic and linear regression analyses.

Outcomes

All-cause in-hospital mortality was the primary outcome of interest. In addition to sepsis, acute kidney injury, sepsis, and peritonitis were the secondary outcomes. Utilizing the appropriate ICD-10-CM/PCS, the incidence of complications was determined. The average hospital costs and LOS were also investigated.

Study analysis

Continuous data are presented as mean with standard deviation and standard error, while categorical data are expressed as frequency and percentage. The Student's t-test was used to analyze continuous variables, while Pearson's Chi-square test was used to compare categorical variables. The primary and secondary outcomes' unadjusted odds ratios were calculated using univariate logistic regression. The final model included a multivariable logistic regression to account for potential confounders. The two-sided p-value cut-off for statistical significance was 0.05. For data analysis, we used STATA® Version 17.0 software. The p-value was set at p < 0.05 for statistical significance. To produce national estimates, all analyses in our study were weighted using the provided discharge weights. The Consumer Price Index was used to adjust the cost of the hospitals for inflation (provided by the U.S. Department of Labor).

## Results

From 2016 to 2018, a total number of 169,245 patients with the diagnosis of AMI were identified. Of these, 10,493 were also diagnosed with ESRD, and 158,752 were not. The mean age of AMI with ESRD and without ESRD was 68 years and 70 years, respectively, in both groups. Additionally, there was a higher incidence of AMI among patients with ESRD in African American (32.1 and 9.2), Hispanic (14.1 and 6.6), Asian (4.0 and 2.0), and Native American (1.0 and 0.3) patients compared to a diagnosis of AMI alone. Caucasians on the other hand had a higher incidence of AMI diagnosis (79.9) compared to both AMI and ESRD (45.8). Teaching hospitals and hospitals in the Midwest and Western regions had a higher incidence of combined AMI and ESRD. Trends also showed a higher incidence of AMI and ESRD for those with Medicare, Medicaid, and Private insurance as opposed to AMI diagnosed without ESRD. Lastly, chronic comorbidities including diabetes mellitus (62 and 28.8), chronic heart failure (45.1 and 18.9), obesity (14.3 and 12.3), and coronary artery disease (51.2 and 31.8) were all associated with a higher incidence of AMI and ESRD compared to AMI alone (Table 1).

**Table 1:** Patients demographics, insurance providers, hospital bed size, region and teaching status, and patient comorbidities comparing the difference in AMI alone with AMI and ESRD.

Patient demographic	ESRD (N=10,493)	Non-ESRD (158,752)	P-value
Mean age	68	70	
Female	52	66	
Race			
Caucasian	45.8	79.9	0.00
African American	32.1	9.2	
Hispanic	14.1	6.6	
Asian	4.0	2.0	
Native American	1.0	0.3	
Others	2.7	1.8	
Insurance			
Medicare	84.6	72.9	0.00
Medicaid	7.4	6.8	
Private	7.4	1.8	
Others/Uninsured	0.5	1.8	
Bed size			
Small	14.8	19.3	0.00
Medium	28.5	30.1	
Large	56.5	50.4	
Hospital Region			
Northeast	20.9	21.0	0.1
Midwest	24.6	22.2	
South	30.0	39.2	
West	17.5	17.2	
Teaching hospital	74.3	66.6	0.00
Chronic comorbidity			
Diabetes mellitus	62.0	28.8	0.00
Chronic heart failure	45.1	18.9	0.00
Obesity	14.3	12.3	0.01
Dyslipidemia	46.2	46.8	0.6
Coronary artery disease	51.2	31.8	0.00

There was a statistically significant difference observed with the in-hospital mortality being increased for those with AMI and ESRD (OR 1.96, 95% CI 01.66-2.32; P = 0.00). There were also higher odds of peritonitis (OR 2.97, 95% CI 2.97-3.91; P = 0.00), sepsis (OR 15.7, 95% CI 13.5-18.26; P = 0.00), and ileus (OR 1.46, 95% CI 1.10-1.95; P = 0.01) in AMI with ESRD compared to non- ESRD group (Table 2). Patients with ESRD in addition to AMI had a significantly longer LOS (7.4 days compared to 5.3, P = 0.00). Similarly, patients with ESRD and AMI had a significantly higher total hospital cost compared to patients with AMI without ESRD ($91,520 compared to $58,175, P = 0.00) (Table 2).

**Table 2:** Prognostic outcomes comparing the difference in AMI alone with AMI and ESRD.

Frequency in %	ESRD (N=10,493)	Non-ESRD (158,752)	OR [95% CI]	P-value
In-hospital mortality	8.5	4.5	1.96 [1.66-2.32]	0.00
Peritonitis	3.1	1.9	2.97 [2.26-3.91]	0.00
Sepsis	7.1	3.8	15.7 [13.5-18.26]	0.00
Ileus	3.1	1.7	1.46 [1.10-1.95]	0.01

## Discussion

The present study provides unique insight into comparing the outcomes of AMI among those with ESRD with those without ESRD using the large NIS database representing 169,245 admissions. This study showed a statistically significant difference in in-hospital mortality in those with AMI and ESRD compared to those without ESRD. In our study, those with both AMI and ESRD also had higher odds of peritonitis, sepsis, and ileus as well as chronic comorbidities including diabetes mellitus, chronic heart failure, obesity, and coronary artery disease. In addition, a higher incidence of combined diagnosis of AMI and ESRD was seen in African American, Hispanic, Asian, and Native American patients, as well as teaching hospitals and hospitals in the Midwest and Western regions. This higher incidence of AMI and ESRD was also observed in those with Medicare, Medicaid, and private insurance. There was no statistically significant difference noted between the two groups for age, gender, bed size, or incidence of dyslipidemia.

According to the Centers for Disease Control and Prevention (CDC), in 2018, about 0.7 million patients (785,883 people) were diagnosed with ESRD [13]. Patients with ESRD on dialysis have an increased risk of AMI [10]. In our study, the incidence of ESRD in those with AMI was 10,493/169,245 (6%), and AMI + ESRD diagnosis was significantly associated with an increased incidence of several complications. This provides evidence that patients with ESRD diagnosis in addition to AMI are associated with an increased incidence of AMI-associated chronic complications and in-hospital mortality. There is still limited research evaluating the impact of ESRD on developing AMI. However, patients with ESRD and ESRD on dialysis are listed as a known risk factor for AMI [9]. Due to altered mineral metabolism, increased inflammation, and uremic toxins, several studies have demonstrated that ESRD patients are at a higher risk for cardiovascular disease, including AMI. Some of the other common risk factors for AMI include old age, diabetes, peripheral vascular disease, atrial fibrillation, heart failure, chronic pulmonary disease, neoplasm, peptic ulcer disease, and peritoneal dialysis [14]. A previous study reported the risk of mesenteric ischemia for ESRD patients is about 44 times higher than that of the general population [10]. Despite the obvious link between AMI and ESRD, very few studies have focused on the impact of ESRD in conjunction with AMI. This study aimed to highlight some of those impacts, and we were able to identify an increased incidence of complications with the combined diagnosis. Additionally, we identified some trends in population, location, and insurance regarding these two groups. More research is needed to further understand the relationship between AMI and ESRD and how the two diseases may impact each other. However, this study successfully addressed the correlation and created a starting point to continue this analysis in the US population.

Our study has several limitations. The temporal sequence of AMI and ESRD was not analyzed as the NIS database does not encode the onset and duration of clinical disorders. The administrative codes used to identify those with these diseases have a high predictive value, but the sensitivity of the codes is unknown at this time, so estimates may not be precise. Additionally, the NIS is not a longitudinal dataset, and the recurrent events may have been counted more than once; however, this would not change our estimation of the overall burden of these disorders. Despite these limitations, our large sample size and data from the hospital across the country are important strengths to note. Large sample sizes can sometimes skew data, making trivial differences appear significant; however, in our case, the large sample size was beneficial- ensuring low confidence intervals, low p-values, and limiting our margin of error. Factors that were found statistically significant based on incidence also seemed to be of practical value. So, we can conclude that in-hospital mortality (8.5% vs. 4.5%) is a statistically and clinically significant complication between the two groups. All other comparisons noted in this research are not contradicted in the literature and are potentially both statistically and clinically significant as well. In addition, we were able to provide the results of a large cohort composed of patients from multiple hospitals, representing all economic and demographic groups as well as all geographical regions of the USA. With this widespread data, we were able to generate results that are generalizable to the whole USA population.

## Conclusions

The current study aimed at understanding how ESRD impacts the prognosis of AMI. This study found that patients with AMI and ESRD had a significantly higher risk for in-hospital mortality compared to patients without ESRD (8.5% compared to 4.5%). Secondly, patients with both AMI and ESRD had higher odds of developing complications like peritonitis, sepsis, and ileus. In patients with ESRD, these complications may be difficult to manage as they are often poor surgical candidates. Overall, this study emphasizes the significance of managing cardiovascular disease risk in ESRD patients, especially in those with an AMI diagnosis. In conclusion, this study offers crucial insights into how ESRD affects the outcome of AMI. It draws attention to the elevated risk of complications and mortality brought on by this comorbid diagnosis. These findings highlight the need for ongoing efforts to improve outcomes in this vulnerable population and have significant implications for clinical practice and research.
